# Clinicopathological findings of a long-term survivor of V180I genetic Creutzfeldt-Jakob disease

**DOI:** 10.1080/19336896.2020.1739603

**Published:** 2020-03-16

**Authors:** Yuichi Hayashi, Yasushi Iwasaki, Masahiro Waza, Shinei Kato, Akio Akagi, Akio Kimura, Takashi Inuzuka, Katsuya Satoh, Tetsuyuki Kitamoto, Mari Yoshida, Takayoshi Shimohata

**Affiliations:** a Department of Neurology, Gifu University Graduate School of Medicine, Gifu, Japan; b Autopsy Center of Prion Disease, Institute for Medical Sciences of Aging, Aichi Medical University, Nagakute, Japan; c Department of Neurology, Kakamigahara Rehabilitation Hospital, Kakamigahara, Japan; d Department of Neurology, Gifu Municipal Hospital, Gifu, Japan; e Department of Locomotive Rehabilitation Sciences, Nagasaki University Graduate School of Medicine, Nagasaki, Japan; f Division of CJD Science and Technology, Department of Prion Research, Center for Translational and Advanced Animal Research on Human Diseases, Tohoku University School of Medicine, Sendai, Japan

**Keywords:** V180I genetic Creutzfeldt-Jakob disease, diffusion-weighted MRI, long-term survivor, pathology, brainstem involvement

## Abstract

The clinical characteristics of genetic Creutzfeldt-Jakob disease (gCJD) with a V180I mutation in the *PRNP* gene (V180I gCJD) are unique: elderly-onset, gradual progression, sporadic fashion, and cortical oedematous hyper-intensity on diffusion-weighted MRI (DW-MRI). This phenotype may become a potential target of future clinical therapeutic trials. The average disease duration of V180I gCJD patients is 23–27 months; however, considerably long-term survivors are also reported. The factors influencing survival and the clinicopathological characteristics of long-term survivors remain unknown. Herein, we report clinicopathological findings of a long-term survivor of V180I gCJD. A 78-year old woman was admitted to our hospital due to dementia and left hand tremor approximately 1.5 months after symptom onset. Neurological examination revealed dementia, frontal signs, and left hand tremor at admission. She had no family history of dementia or other neurological disease. DW-MRI revealed cortical oedematous hyper-intensities in the bilateral frontal lobes and the right temporal and parietal lobes. *PRNP* gene analysis indicated a V180I mutation with methionine homozygosity at codon 129. The symptoms gradually progressed, and she died of aspiration pneumonia 61 months after symptom onset. Neuropathological examination revealed severe cerebral atrophy with moderate to severe gliosis, but the brainstem was well preserved. Various-sized and non-confluent vacuole type spongiform changes were extensively observed in the cerebral cortices. Prion protein (PrP) immunostaining revealed weak and synaptic-type PrP deposits in the cerebral cortices. We consider that long-term tube feeding, and very mild brainstem involvement may be associated with the long-term survival of our V180I gCJD patient.

## Introduction

Genetic Creutzfeldt-Jakob disease (gCJD) with a V180I mutation (V180I gCJD) in the *PRNP* gene is the most common type of gCJD in Japan, and accounts for 41.2% of gCJD patients [1]. Conversely, V180I gCJD is extremely rare in European countries, the United States, and China [2–4]. Its clinical characteristics are unique: elderly-onset, gradual progression, sporadic fashion, and cortical oedematous hyper-intensity on diffusion-weighted MRI (DW-MRI) [5,6]. Moreover, we previously reported unique single-photon emission computed tomography patterns such as preserve cerebral blood flow in the occipital cortices, brainstem, and cerebellum within the initial 2–3 years after disease onset [7]. The average disease duration of V180I gCJD patients is 23 or 27 months in methionine homozygote or M/V heterozygote at codon 129 in the *PRNP* gene, respectively [6]. Long-term survivors whose disease duration is ≧ 5-10 years have also been reported [8,9]; however, the influencing survival and the clinicopathological characteristics of long-term survivors of V180I gCJD remains unknown. Herein, we report the clinicopathological findings of a long-term survivor of V180I gCJD, and consider for a cause of the long-term survival.

## Patient and methods

### Clinical summary

**Figure 1.**
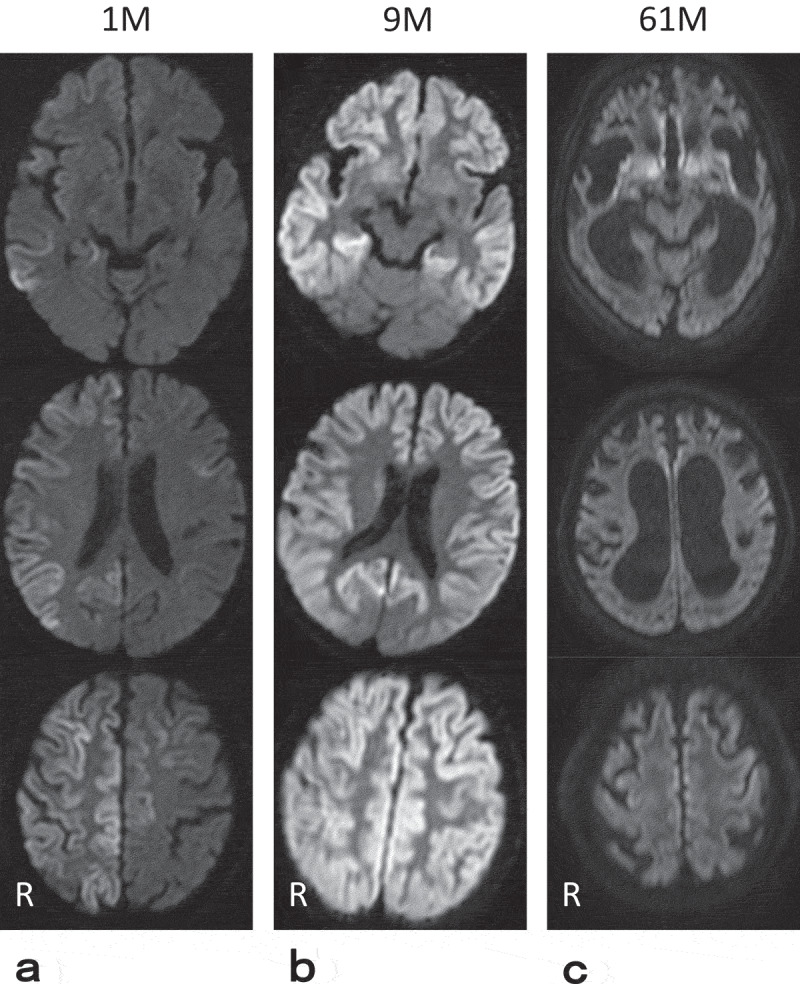
Serial diffusion-weighted MR images. Diffusion-weighted MR images (DW-MRI) obtained from 1.5 (a), 9 (b), or 61 (c) months after disease onset, respectively. Panel aillustrates the cortical oedematous hyper-intensities observed predominantly in the right frontal and temporal lobes excluding basal ganglia, at 1.5 months after onset (a). DW-MRI performed 9 months after the onset exhibited increased signals compared to those on images acquired 1.5 months after onset; the affected area demonstrated bilateral expansion to the basal ganglia and parietal lobes (b). DW-MRI obtained 61 months after onset revealed that the cerebral atrophy had significantly progressed, and both oedema and signal intensities in the bilateral cortices were diminished compared to those on images taken 9 months after onset; however, hypertensive areas were observed in the bilateral basal ganglia (c).

A 78-year-old Japanese woman was admitted to our hospital due to a 1.5-month history of cognitive impairment and tremor in the left hand, without any family history of these symptoms. Neurological examination revealed moderate cognitive impairment (15/30 points of the MMSE), left hand-tremor, bradykinesia, and parkinsonian gait, as described previously [7]. Cerebrospinal fluid (CSF) analysis revealed elevated levels of 14-3-3 protein and normal total tau protein levels. However, the prion proteins (PrP) in the CSF were not amplified by the real-time quaking-induced conversion method [10]. *PRNP* gene analysis revealed a V180I mutation with methionine homozygosity at codon 129. DW-MRI revealed cortical oedematous hyper-intensities in predominantly the right frontal and temporal lobes, excluding the basal ganglia, at 1.5 months after the onset (Figure 1a). We diagnosed the patient with V180I gCJD. Forced laughing was observed 9 months after the onset of symptoms; however, neither the startle response nor forced crying was observed during the disease course. DW-MRI acquired 9 months after the symptom onset revealed increased signals compared to the images taken 1.5 months after onset; the affected area exhibited bilateral expansion to the basal ganglia and parietal lobes (Figure 1b). Her symptoms gradually deteriorated to an akinetic mutism state by 14 months after onset, and she underwent tube feeding. The patient transferred to a chronic care hospital. Thereafter, we underwent long-term follow-up by our multidisciplinary medical network team for prion disease until death [11]. The symptoms gradually progressed, and the patient died of aspiration pneumonia 61 months after onset. DW-MRI performed 61 months after onset revealed that the cerebral atrophy had significantly progressed, and both the oedema and signal intensities in the bilateral cortices were diminished compared to those in images acquired 9 months after onset; however, hypertensive areas were observed in the bilateral basal ganglia (Figure 1c). We obtained informed consent to perform an autopsy from her family, and we transferred the patient’s body to the Autopsy Centre of Prion Disease.

### Neuropathological examination

A post-mortem study was performed 22.5 h after death. The brain, and upper cervical spinal cord were fixed in 20% neutral-buffered formalin for 4 weeks, and tissue blocks were immersed in 95% formic acid for 1 h to inactive prion infectivity. The specimens were then embedded in paraffin and cut into 4-μm sections. The sections were deparaffinized in lemosol, rehydrated through an ethanol gradient, and stained. For routine neuropathological examinations, sections were subjected to haematoxylin-eosin, Klüver-Barrera’s (KB) and modified Gallyas-Braak silver stainings. Referring to the Iwasaki’s pathological staging for sporadic CJD (sCJD) [12], we evaluated the severity of the neuropathological changes as mild, moderate or severe.

Immunohistochemical analysis was performed with a monoclonal antibody against PrP (3 F4; Dako, Glostrup, Denmark, mouse monoclonal, diluted 1:100) after hydrolytic autoclaving for antigen retrieval [13]. PrP immunostaining was conducted as previously described [14]. Immunostaining with anti-Aβ (4G8; Signet. Dedham, MA, mouse monoclonal, diluted 1:2,000), anti-hyperphosphorylated tau (AT-8; Innogenetics, Ghent, Belgium, mouse monoclonal, diluted 1:1,000), and anti-phosphorylated α-synuclein (pSyn#64; Wako Pure Chemical Industries, Osaka, Japan, mouse monoclonal, 1:3,000) was also performed. In these immunostainings, primary antibody binding was detected using the envision amplified visualization method (En Vision plus kit; Dako). Peroxidase-conjugated streptavidin was visualized with 3, 3ʹ-diaminobenzidine (DAB; Wako Pure Chemical Industries) as the final chromogen. Immunostained sections were lightly counterstained with Mayer’s haematoxylin.

### Western blot analysis of protease-resistant PrP

The cryopreserved right frontal cerebral cortex, which was snap frozen and stored at −80°C prior to use, was homogenized, and Western blot analysis of protease-resistant PrP was performed using 3F4 antibodies, as previous described [14].

## Results

### Macroscopic findings

The patient’s brain weighed 920 g. Macroscopic analysis revealed severe diffuse cerebral atrophy predominantly in the frontal and temporal lobes. No apparent atrophy was observed in the brainstem and cerebellum.

### Microscopic findings

**Figure 2.**
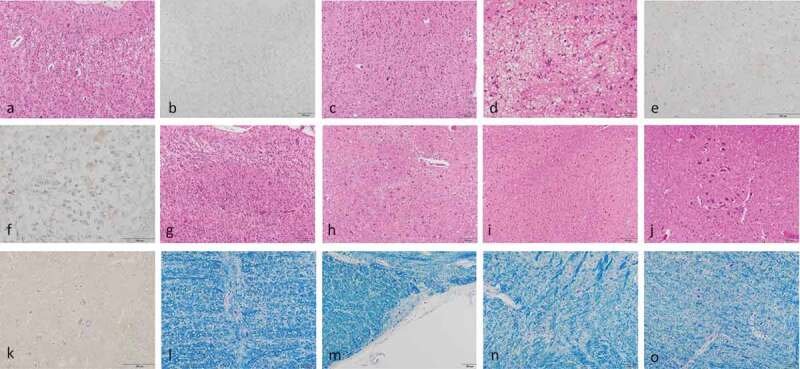
Microscopic findings. Haematoxylin-eosin stains (A, C, D, G, H, I, J); anti-prion protein (PrP) immunostains using 3F4 antibodies (B, E, F, K); Klüver-Barrera’s (KB) stains (L, M, N, O); the middle frontal gyrus (A, B); the precentral gyrus (C); the cortex of the amygdala (D, E); the subiclum (F); the striate area of the posterior lobe (G); the cerebellar dentate nuclei (H); the inferior olivary nuclei (I); the C3 level of the spinal cord (J, K); the obscurus raphe nuclei (L), accurate nuclei of medulla oblongata (M), pre-Bötzinger complex (N), and dorsal respiratory group of the medulla (O).Moderate spongiform changes with neuropil rarefaction were extensively observed in the cerebral cortex, and the amygdala (a, d). The morphology of the spongiform changes showed various-sized and non-confluent vacuoles (a, d). Severe gliosis and neuronal loss were recognized. However, the spongiform changes in the precentral gyrus and the cortices of the occipital lobes were milder than in those of frontal and temporal lobes (c, g). The spongiform changes and gliosis were mild in the cerebellum dentate nuclei, inferior olivary nuclei, and upper cervical spinal cord (h, i, j). Anti-PrP immunostains were generally weak in the neocortices (b), amygdala (e), the subiclum (f), or upper cervical spinal cord (k). KB stains revealed preserved neurons in the obscurus raphe nuclei (l), accurate nuclei of medulla oblongata (M), pre Botzinger complex (N), and dorsal respiratory group of the medulla (o).

Moderate spongiform changes with neuropil rarefaction were extensively observed in the cerebral cortex, striatum and amygdala (Figure 2a,d). Morphologically, the spongiform changes consisted of various-sized and non-confluent vacuoles (Figure 2a,d). Severe gliosis and neuronal loss were recognized. Mild hypertrophic astrocytosis and ballooned neurons were also noted. However, the spongiform change in the precentral gyrus and the cortices of the occipital lobes were relatively milder than in those of the frontal and the temporal lobes (Figure 2c,g). The hippocampal formation, subiculum and parahippocampal gyrus were generally preserved with mild spongiform changes. The globus pallidus, lateral thalamus, and subthalamic nucleus were generally unaffected. The cerebral white matter exhibited mild myelin pallor with apparent gliosis. The spongiform changes and gliosis were mild in the cerebellar dentate nuclei, inferior olivary nuclei, and upper cervical spinal cord (Figure 2h–j). The neurons were preserved in the raphe obscurus nuclei, accurate nuclei, pre-Bötzinger complex, and dorsal group in the KB stains (Figure 2l–o). In general, the brainstem, cerebellum and spinal cord were not severely involved as compared with cerebrum in this patient.

### PrP immunohistochemical findings

PrP immunostaining revealed diffuse synaptic-type PrP deposits in the cerebral cortex, cerebellar cortex, and anterior and posterior horns of the spinal cord, but the immunoreactivity was generally weak (Figure 2b,e,f,k). PrP deposits were few in number in the medial temporal cortex and were partially enhanced in the senile plaques, particularly in the parahippocampal gyrus. No PrP deposits were observed in the cerebral white matter or brainstem.

### Ageing and accompaniment pathological findings

**Figure 3.**
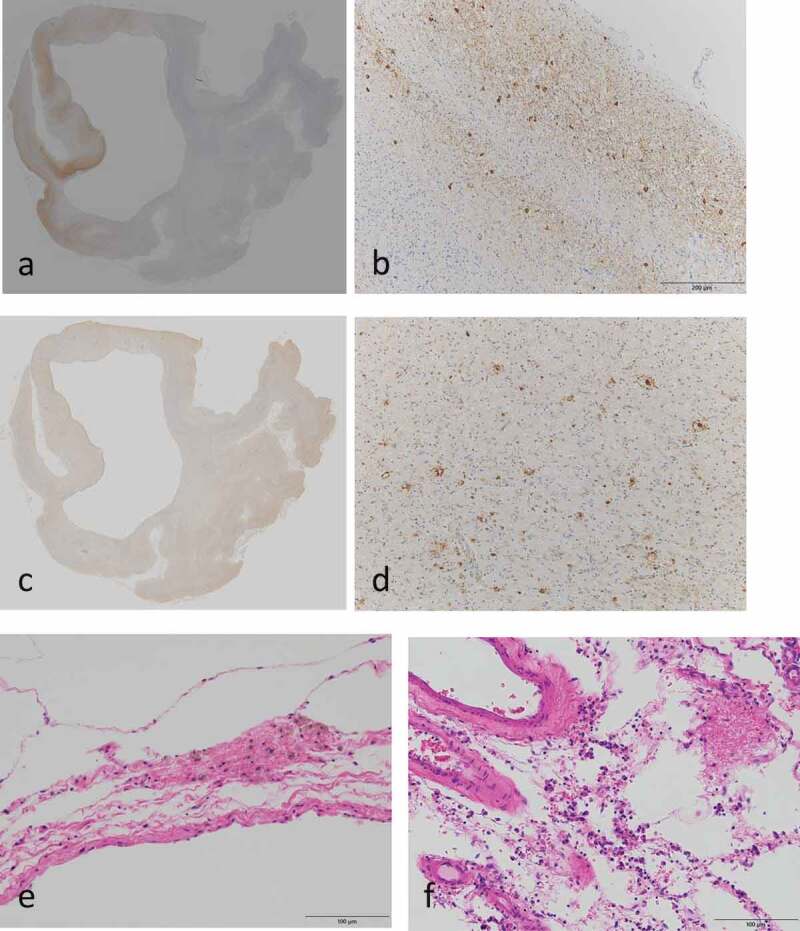
Ageing and accompaniment pathological findings. AT8 stains (a, b); Aβ stains (c, d); Haematoxylin-eosin stains (e, f); the hippocampus (a, c); the entorhinal cortex (b); the calcarine sulcus (d); pia matter of the superior parietal lobule (e) and of the calcarine sulcus (f). Multiple micro-abscesses and inflammation changes in the pia matter were found (e, f)

Ageing pathology was moderately observed. Neurofibrillary tangles (NFT) were found in the parahippocampus and entorhinal cortex and were determined to be Braak NFT stage II or AT8 NFT stage II (Figure 3a,b) [15]. The senile plaques were determined to be CERAD B, Braak stage B [15], and Thal phase 3/4 (Figure 3c,d) [16]. Alzheimer’s disease pathology was mild according to the NIA-AA criteria [17]. Amyloid angiopathic change was evident. Argyrophilic grain was mild and was determined to be the Saito stage 1 [18]. Tufted astrocytes and astrocytic plaques were not found. Brain calcification was not found in the basal ganglia.

Multiple micro-abscesses and inflammation changes in the pia matter were found in the cerebrum, cerebellum, brainstem and spinal cord (Figure 3e,f). These infectious changes were secondary caused by severe aspiration pneumonia and the terminal stage of sepsis.

### Findings of the Western blot analysis

**Figure 4.**
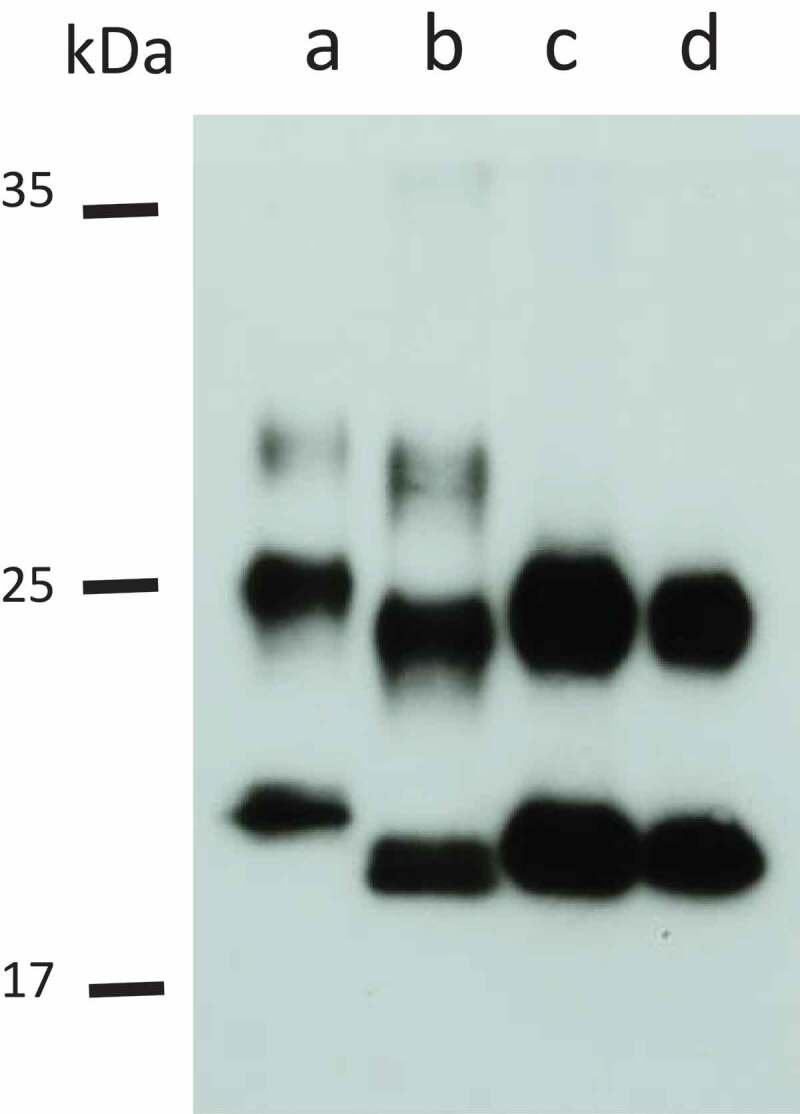
Western blot analysis of protease K-resistant prion protein. Lane **a**: a control sample from the MM1 type of a sporadic Creutzfeldt-Jakob disease (sCJD) patient (6 fold diluted); lane **b**: a control sample from an MM2-cortical type of sCJD patient (25 fold diluted); lane **c**: a sample from the present patient (undiluted); lane **d**: a control sample from a V180I genetic Creutzfeldt-Jakob (gCJD) patient (undiluted). A characteristic glycoform pattern typical of V180I gCJD was observed in the current patient (**c**). No diglycoform band (upper glycoform) was detected. The molecular weight of the non-glycoform band (unglycosylated) was consistent with type 2 prion protein, but was slightly higher than that characteristic of sCJD (**c**).

The characteristic glycoform pattern of V180I gCJD [19] was also observed in the current patient. No diglycoform band (upper glycoform) was detected. The molecular weight of the non-glycoform band (unglycosylated) was consistent with type 2 PrP, but was slightly higher than that characteristic of sCJD (Figure 4).

## Discussion

We herein report a patient with V180I gCJD who survived for more than 5 years after disease onset. We focused on two clinicopathological characteristics associated with long disease duration: appropriate long-term care for dysphagia, and mild involvement of the brainstem.

First, we described the clinical findings of the patient. This patient presented with severe pseudobulbar paralysis by 14 months after the onset; thereafter, she received long-term tube feeding. However, respiratory and cardiac functions were preserved during her disease course. A previous report indicated that the survival period of tube-fed patients with sCJD is longer than that of patients who were not tube fed [20]. To understand the timing of starting tube-fed or total parenteral nutrition (TPN), we reviewed 14 patients with autopsy-confirmed V180I gCJD including the current patient (Table 1) [8,21–33]. Combined V180I and M232R gCJD patients were excluded from our review. The clinical information for receiving tube-fed or TPN was available for only four patients. Three patients received tube-fed, and one patient received TPN in the late stage of the disease. Average (range) months between symptom onset and starting tube-fed or TPN, or duration tube-fed or TPN were 19.3 (11–30), or 33 (2–80) months, respectively. Our patient also received appropriate long-term care with tube feeding, and this could be associated with her long disease duration.

**Table 1.** Clinicopathological findings of autopsy-verified V180I genetic Creuztledt-Jakob disease patients except for V180I/M232R combined patients.

Number	Author (y)	Onset of age (y)	Death of age (y)	Sex	Disease duration (mo.)	Initial symptom	Tube-fed or TPN (duration between onset and starting tube-fed orTPN) (mo.)	Duration tube-fed or IVH (mo.)	Duration between onset and akinetic mutism
1	Matsumura (1995) [21]	77	79	F	25	Rt. Tremor	N.D.	N.D.	18
2	Iwasaki (1999) [22], Akagi (2018) [8]	80	82	M	21	Rt. hemiparesis, motor aphasia	Oral intake until death	N.D.	9
3	Chassigneaux (2006) [23]	66	69	M	54	Dementia	N.D.	N.D.	12
4	Shindo (2006) [24]	79	84	F	61	Dementia, bradykinesia	N.D.	N.D.	N.D.
5	Suzuki (2008) [25], Suzuki (2009) [26]	79	80	M	13	Dementia	TPN (11)	2	+ (N.D.)
6	Tsuboi (2009)[27], Honda (2013) [28]	64	70	F	72	Disorientation, memory deficit	N.D.	N.D.	N.D.
7	Yoshida (2010) [29]	77	79	F	26	Gait instability	N.D.	N.D.	18
8	Iwasaki (2011) [30], Akagi (2018) [8]	73	81	F	102	Aphasia	Tube-fed (22)	80	22
9	Yeo (2013) [31]	75	75	F	0.17	Semicoma	N.D.	N.D.	-
10	Iwasaki (2017) [32], Akagi (2018) [8]	78	81	F	33	Disorientation	Tube-fed (30)	3	16
11	Iwasaki (2018) [33], Akagi (2018) [8]	87	87	F	10	Slow reaction	Oral intake until death	N.D.	-
12	Akagi (2018) [8]	84	85	F	20	Numbness, tremor	N.D.	N.D.	5
13	Akagi (2018) [8]	73	81	F	101	Disorientation	N.D.	N.D.	20
14	Current patient	78	83	F	61	Lt. tremor, dementia	Tube-fed (14)	47	14
	AVG + SD [range]	76.4 ± 6.1 [64–87]	79.7 ± 5.2 [69–87]	F (11/14)	42.8 ± 32.8 [0.17–101]		19.3 ± 8.5 [11–30] (n = 4)	33.0 ± 37.7 [2–80] (n = 4)	14.9 ± 5.5 (n = 9)

Cause of death	Cortical hyperintensities on initial DW-MRI	Initial DW-MRI perfoemd after the onset (mo.)	CSF 14-3-3 protein (μg/ml)	CSF total tau-protein (pg/mL)	Polymorphism at the codon 129 in the *PRNP gene*	Brain weight (g)	Cerebral cortical involvement	Pathological brainstem involvment
Penumonia	N.E.	N.E.	N.D.	N.D.	M/V	1220	+	N.D.
Pneumonia	+	10	N.E.	N.E.	M/V	1060	+	Absent
N.D.	N.D.	N.D.	N.D.	N.D.	M/M	N.D.	+	N.D.
Respiratory failure	+	6	+	N.D.	M/V	N.D.	+	Absent
Lung abscess, Pneumonia	+	N.D.	+	N.D.	M/V	1180	+	N.D.
Pneumonia	+	N.D.	N.D.	N.D.	M/M	484	+	relatively preserved from atrophy, PrP depositts and microspheres in the midbrain
Pneumonia	+	N.D.	+	N.D.	M/M	950	+	Absent
Respiratory failure	+	42	+ (3423)	5474	M/M	720	+	Absent
Cardiovascular collapse	+	12 h	+	N.D.	M/M	N.D.	+	Absent
Respiratory failure	+	8	+ (1435)	1370	M/M	750	+	Absent
Respiratory failure	+	1	N.E.	N.E.	M/M	1050	+	Absent
Respiratory failure	+	4	+ (697)	>1200	M/M	1150	+	Absent
Respiratory failure	N.E.	N.E.	N.E.	N.E.	M/M	600	+	Absent
Pneumonia, sepsis	+	1.5	+	-	M/M	920	+	Very mild involvement
	+ (11/12)	9.1 ± 13.8 (n = 8)	+ (8/8)	+ (3/4)	M/M (10/14)	960 ± 212 (n = 12)	+ (14/14)	Absent or very mild (10/11)

N.D.: not described; N.E.: not examined; DW-MRI: diffusion-weighted MR imge, PrP: prion protein; y: year-old; mo.: months; h: hours; F: female; M: male; +: positive; -: negative; Rt. Right; Lt.Left.

Second, the pathological characteristics of the current patient included moderate spongiform changes, severe gliosis, and severe neuronal loss in the cerebral cortices. However, the brainstem involvement was very mild. To understand the absence or presence of brainstem involvement, we reviewed 14 patients with autopsy-confirmed V180I gCJD including the current patient (Table 1). The pathological findings in the brainstem were available for 11 of the 14 patients with V180I gCJD (Table 1). Ten of these 11 patients had mild or no involvement of the brainstem, regardless of disease duration. The remaining patient, who had received continuous intraventricular pentosane polysulfate infusion therapy, had numerous microspheres as well as PrP synaptic depositions in the central grey matter of the midbrain; however, medullar involvement was not described [28]. Moreover, Akagi, *et al* reported that the long-term survivors exhibited moderate spongiform changes regardless of disease duration, severe gliosis, and severe neuronal loss in the post-mortem analysis, and short-term survivors exhibited severe spongiform changes, moderate gliosis, and mild neuronal loss in the cerebral cortices [8]. The pathological findings of the current patient revealed a pathological pattern typical of a long-term survivor. Thus, we consider that two clinicopathological characteristics associated with long disease duration exist: appropriate long-term care with tube feeding, and very mild involvement of the brainstem with moderate cerebral spongiform changes. Especially, our observational study shows pseudobulbar palsy is a main cause of dysphagia in a patient with V180I gCJD, and an appropriate long-term care with tube feeding is crucial for a long-term follow-up

To date, despite several clinical trials, no effective treatment has been discovered for human prion disease. The rarity, rapidity, and clinical heterogeneity of prion disease affects study enrolment and the ability to measure treatment outcomes [34]. Since V180I gCJD is a gradually progressing prion disease [6], we believe that this phenotype may become a potential target of clinical therapeutic trials for prion disease as well as MM2-cortical-type of sCJD [35].Therefore, in the setting of clinical trials, a good understanding of the clinicopathological characteristics of a long-term survivor of V180I gCJD is required.
